# Evaluation of a Simple Antibiotic-Free Cryopreservation Protocol for Drone Semen

**DOI:** 10.3390/insects16010050

**Published:** 2025-01-07

**Authors:** Sophie Egyptien, Jérôme Ponthier, Fabien Ectors, Brice Thibaut, Stéfan Deleuze

**Affiliations:** 1 Fundamental and Applied Research for Animals and Health Research Unit (FARAH), Comparative Veterinary Medicine, Faculty of Veterinary Medicine, University of Liège, 4000 Liège, Belgium; 2 Fundamental and Applied Research for Animals and Health Research Unit (FARAH), Veterinary Public Health, Faculty of Veterinary Medicine, University of Liège, 4000 Liège, Belgium; 3 Independent Researcher, 4000 Liège, Belgium

**Keywords:** honey bee, drone semen, cryopreservation, antibiotic, viability, *Apis mellifera*, instrumental insemination

## Abstract

The increasing reliance of modern agriculture on honey bee (*Apis mellifera*) pollination has driven efforts to preserve and enhance bee populations. The cryopreservation of drone semen presents a promising solution for preserving genetic diversity and supporting breeding programs without live animal transport risks. This study aimed to evaluate a one-step dilution antibiotic-free drone semen slow-freezing protocol under field conditions with in vitro and in vivo parameters. Semen viability was tested by two different mixes of dyes, and both techniques gave similar results, showing a post-thaw viability drop of 37%. Virgin queens were inseminated either with fresh or frozen–thawed semen. Survival rates until egg-laying onset and female brood production were similar for both groups; however, colonies with queens inseminated with fresh semen were more likely to go into wintering. Results suggest that frozen–thawed semen can support viable queen insemination, with potential for female brood production even without antibiotics in the diluent. This study highlights the need for further refinement of cryopreservation protocols, particularly regarding semen quality and queen longevity, to improve the feasibility of cryobanking for *Apis mellifera* conservation and breeding.

## Simple Summary

Modern agriculture relies mainly on the honey bee for pollination. Therefore, it is crucial to preserve and increase bee populations. Breeding programs have a crucial role, and insemination with frozen semen bypasses many practical limitations. This study evaluates the effectiveness of a simple antibiotic-free freezing protocol by assessing semen viability using two different tools and female brood production. The drop in viability associated with cryopreservation is 37%, and both evaluation tools give similar results. Despite that loss, 5/8 queens inseminated with frozen–thawed semen produced female brood, which is similar to the results obtained with the control group inseminated with fresh semen. This demonstrates that cryopreserved semen following this protocol is capable of egg fertilization. That, in turn, is very promising as this is the first antibiotic-free protocol reported in the literature to prove effective in female brood production.

## 1. Introduction

The agricultural industry has become highly dependent on pollinators in recent decades, with *Apis mellifera*, the domesticated honey bee, playing a central role in crop pollination [1].

In many places, the growing demands of agriculture are no longer met [1] because of an insufficient global increase in the number of colonies and localized declines in populations, particularly in North America and several European countries [2].

Given their crucial role in pollination, it is essential to develop and improve methods for preserving the domesticated honey bees, along with subspecies and lineages adapted to diseases and predators. Preserving their genetic material can be achieved by maintaining live colonies or conserving live cells. Semen from drones, the haploid males, represent valuable cells to cryopreserve as a genetic safeguard.

Cryobanking of drone semen contributes to the preservation and restoration of colonies from known lineages, thus maintaining biodiversity. It supports breeding programs by allowing the shipping of semen worldwide, as the exchange of cryopreserved semen has proven to be a safe and effective [3] alternative to the transportation of live animals, which may be complex and often illegal at an international scale. Although proven effective, the transport of live material must always be carried out with caution as the container and the semen might represent vectors for infectious agents, such as bacteria or viruses, like in other species [4,5]. Cryopreserved semen can also be used for inseminations during periods when fresh semen is unavailable.

Slow-freezing protocols yielding satisfactory results have been reported on different occasions. Most of these involved several steps, including one or two dilutions, pre- or post-freezing centrifugation, and/or post-thaw cryoprotectant removal [6,7,8,9,10,11,12], with only a few [13,14,15,16,17,18] being single-step procedures. Some of the reports have evaluated post-thaw semen quality by in vitro techniques only [10,11,12,15,16,18], while others ran fertility trials to further confirm post-thaw quality [6,7,8,9,13,14]. All of the published protocols used antimicrobial agents. In the context of antimicrobial resistance, the non-therapeutic use of antibiotics, like in semen extenders, has become a matter of concern in many domestic species [19]. To the best of our knowledge, the production of female brood after insemination with an antibiotic-free frozen thawed drone semen antibiotic-free protocol has yet to be reported.

The most common semen quality parameter evaluated is viability. It is defined by the permeability of the plasma membrane: viable cells have an intact, impermeable plasma membrane [20]. Viability assessment is mostly performed under fluorescent microscopy using DNA markers such as propidium iodide (PI), which marks non-viable cells in red associated with SYBR-14 (SYBR) or Hoechst 33342 (Hoechst), both able to cross cell membranes regardless of their integrity [21]. Recently, Calcein Violet 450 AM (CaV), a dye marking the cytoplasm of cells with an intact membrane, has been suggested as a useful alternative to SYBR and Hoechst in several species, including drones [22].

The aim of this study is to evaluate a simple (one-step dilution) antibiotic-free protocol to freeze drone semen in vitro by comparing two combinations of dyes to assess post-thaw semen viability and in vivo by inseminating queens in a fertility trial.

## 2. Materials and Methods

The protocol was conducted in triplicate during the spring and summer of 2024.

### 2.1. Drone Semen Collection

Semen was collected once a month from May to July 2024 at the apiary using a Harbo syringe (Harbo, 1985) 9 to 16 days before freezing or insemination. Drones were collected until the glass straw was full (±150 µL). The glass capillary tube was then sealed and kept in the dark at 16 °C as described in standard procedures [8,12]. The three replicates of semen collection are referred to as 1, 2, or 3 hereafter.

### 2.2. Semen Freezing and Thawing

The day the semen was frozen, the semen sample was homogenized, 50 µL was loaded into a new sterile glass straw for insemination planned the next day, and 50 µL was prepared for freezing.

The dilution and straw-filling process were adapted from a previously published protocol [8]. Briefly, 50 µL of fresh semen was mixed with 33 µL of a cryopreservation diluent, referred to as Harbo’s diluent, which consisted of 500 µL of buffer (NaH_2_PO_4_ and Na_2_HPO_4_, pH 7.21, ThermoFisher, Waltham, MA, USA), 250 µL of fresh egg yolk, and 250 µL of DMSO (ThermoFisher, Waltham, MA, USA). A 250 µL plastic straw was then filled with layers as follows: 20 µL of cryoprotectant, an air bubble, the diluted semen, another air bubble, and, finally, another 20 µL of cryoprotectant. One end was sealed with cotton and gelatin, while the other was closed using a manual ultrasonic sealer (CryoSealer, Minitüb GmbH, Tiefenbach, Germany).

The straw was then placed in a controlled-rate liquid nitrogen freezer (Freeze Control^®^ Cryopreservation System, Cryologic Pty. Ltd., Victoria, Australia) with the following freezing protocol: starting at room temperature, the temperature was lowered at a rate of −1 °C per minute to 5 °C, held for 1 min, then reduced at −2 °C per minute to −6 °C, held for 1 min with ice seeding (to activate crystallization), and then at −5 °C per minute to −40 °C, before finally being plunged into liquid nitrogen and stored. The next day, semen was prepared for insemination and thawed in water at 35 °C for 30 s. Semen was expelled into a PCR tube, a sample of 2 µL was used for post-thaw semen analysis, and the rest was mounted in a new sterile glass capillary for insemination.

### 2.3. Instrumental Insemination

Twenty-nine queens, aged 7 to 12 days, were anesthetized with CO_2_ the day before insemination for 3 min to stimulate oviposition and were inseminated following a standard method [8]. Insemination was conducted under CO_2_ anesthesia and completed within 5 min of induction. A Pierre Marin insemination syringe (Pierre Marin, Forchies-la-Marche, Belgium) was used under a microscope to deposit 5 µL of either fresh (F) or frozen–thawed (Fz) semen in 13 and 16 queens, respectively. Three insemination sessions, each including both F and Fz semen, took place one month apart from May to July 2024, corresponding to the replicates of semen collection 1, 2, or 3.

Immediately after insemination, the queens were caged and placed in queenless nucleus hives with young bees (Mini-Plus hive, Stehr, Schwalmtal, Germany), where they remained for the duration of the experiment.

### 2.4. Brood Evaluation and Third Narcosis

Five days after introducing the queens into the hives, each hive was examined to confirm queen acceptance (release from her cage by worker bees) and the onset of egg laying. If a queen was accepted but had not begun egg laying within 11 days of insemination, she was subjected to a second round of CO_2_ treatment to stimulate egg production [8]. Capped brood evaluations were performed every 3 to 5 days starting one week after egg observation, monitoring for the presence of female larvae.

### 2.5. Semen Analysis

When insemination doses were prepared, semen analysis was performed by the same operator for consistency, with fresh and frozen–thawed samples diluted at 1:200 in phosphate-buffered saline (PBS) before assessment.

#### 2.5.1. Semen Concentration

Concentration was measured using a hemocytometer (Thoma cell counting chamber, Marienfeld^®^, Lauda-Königshofen, Germany). A 10 µL diluted semen sample diluted in 390 µL of 2% formaldehyde was used for counting. Spermatozoa counts from 10 squares of 0.2 mm were obtained under an optical microscope at 400× magnification. The concentration of undiluted semen was calculated using the formula: (number of sperm heads/0.04) × 40 × 200 = concentration in millions of spermatozoa/µL.

#### 2.5.2. Motility

A 3 µL drop of diluted semen was placed in a pre-warmed slide (Leja, IMV technologies, Saint-Ouen-sur-Iton, France), and motility was grossly assessed after 5 min using brightfield optical microscopy. The microscope stage was kept at 37 °C, and motility was noted if any movement was observed.

#### 2.5.3. Viability

Viability was assessed using fluorescent microscopy, and dyes were used following manufacturer recommendations and a previously described protocol [22]. The first fluorescent dyes mix (Mix 1) consisted of SYBR14 (SYBR) and propidium iodide (PI) from the LIVE/DEAD™ Sperm Viability kit, with the addition of Hoechst 33342 (Hoechst), which binds to the DNA of all cells and fluoresces in blue. The Hoechst stock solution was diluted 1:99 before staining 50 µL of the semen sample with 1 µL. When associated with PI, SYBR stains live sperm cells in green, and PI highlights dead cells in orange-red. The second mix of dyes (Mix 2) was composed of Draq5^TM^ (Draq5), Calcein Violet 450 AM (CaV), and PI. Draq5 is a far red dye with the same properties as Hoechst. All dyes were sourced from ThermoFisher (Waltham, MA, USA). Images of spermatozoa were captured with an Observer 7 widefield fluorescent microscope (Zeiss, Oberkochen, Germany) equipped with a 40×/1.1 NA oil immersion objective. The samples were illuminated using wavelengths of 385 nm, 385 nm, 475 nm, 555 nm, and 653 nm for Hoechst, CaV, SYBR, PI, and Draq5, respectively. Brightfield imaging was also used. Emission wavelengths were filtered as follows: 430–470 nm for Hoechst and CaV, 500–550 nm for SYBR, 570–640 nm for PI, and 665–715 nm for Draq5. Images were automatically overlayed using Zen Blue 2.6 software (Zeiss, Oberkochen, Germany). The initial focus was achieved using the 430–470 nm emission filter in Mix 1 and 665–715 nm in Mix 2.

All samples were included in the analysis, regardless of semen quality and potential fertility.

## 3. Results

### 3.1. Semen Analysis

**Figure 1:**
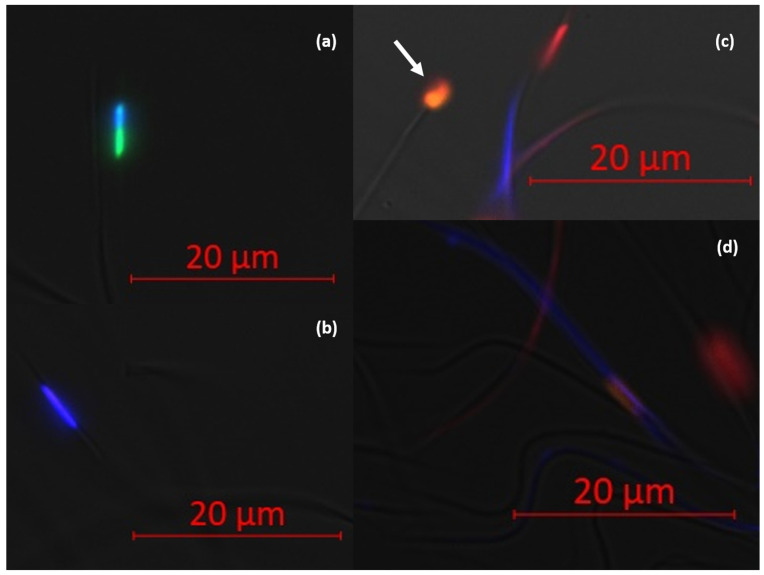
Fluorescence microscopy of sperm stained with Mix 1: Hoechst, SYBR, and PI (**a**,**b**), and Mix 2: Draq5, CaV, and PI (**c**,**d**). (**a**) The sperm cell is viable. The nucleus is stained with Hoechst (blue) and SYBR (green), indicating an intact membrane. (**b**) The sperm cell is non-viable. The nucleus is stained with Hoechst (blue) and PI (orange), indicating a damaged cell membrane. (**c**) The sperm cell marked by the arrow is non-viable. The nucleus is stained with Draq5 (red) and PI (orange). The second sperm cell is viable. The nucleus is stained with Draq5 and the cytoplasm with CaV (blue), indicating an intact cell membrane. (**d**) The sperm cell is moribund, its nucleus is stained with Draq5 (red) and PI (orange), and its cytoplasm is stained with CaV (blue).

Semen analysis showed variability across replicates. Two of the three batches exhibited reduced viability after thawing. Viability results varied with the mix of dyes used. Mix 2 depicted lower viability when the number of cells evaluated was lower than 100. No moribund cell (SYBR and PI positive) was spotted with Mix 1 compared to Mix 2 where the percentage of moribund (CaV and PI positive) cells varied from 0 to 4% (F1: 2%; Fz1: 4%; F2: 2%; Fz2: 3%; F3: 0%; Fz3: 4%). Semen concentration in fresh samples ranged from 6.4 to 15.4 million sperm/µL, whereas frozen–thawed samples ranged from 4 to 6 million sperm/µL, affecting the number of sperm inseminated per queen. When technical issues allowed (Mix 1), the mean viability was 85% and 48% for F and Fz semen, respectively. Table 1 details the semen analysis results, including viability, concentration, total sperm cells per queen, the number of live sperm cells inseminated, and motility. Figure 1 illustrates sperm cells stained with Mix 1 and Mix 2.

### 3.2. Insemination Outcomes

The post-insemination average weight of the queens was 169.41 ± 0.02 mg. The queens’ survival and acceptance rates were similar between groups. Some queens did not survive release, were not accepted by their colonies, or failed to lay eggs. Among surviving queens that laid eggs (five F and eight Fz), three from the F group and five from the Fz group produced female brood. One queen from the Fz1 group, after initially producing female brood, was quickly replaced by worker bees. The new queen mated naturally and went on to produce a typical proportion of female brood. Outcomes were similar in both groups in terms of survival until egg laying and female brood production. Subjectively, the three F colonies and one from the Fz group were considered strong enough for overwintering, although at the time of the last examination before winter, the Fz was not as strong as the F colonies. Detailed insemination outcome data are provided in Table 2.

## 4. Discussion

Except for one report [14], female brood production following inseminations with frozen–thawed semen is less effective than after insemination with fresh semen. It is commonly accepted that the primary objective of insemination with frozen–thawed semen is to produce queens capable of generating sufficient fertilized eggs to establish new, healthy queens, thereby preserving genetic lines and supporting selective breeding [6]. It may be considered that the female brood produced from Fz was of good quality as one colony replaced the queen by rearing a new one from her fertilized egg. This new queen was naturally mated and thrived normally until the colony was robbed out six weeks later.

There are few studies on in vivo outcomes of inseminations using frozen–thawed semen and even fewer examining brood characteristics [6,7,8,9,23] despite this being one of the most accessible and essential field parameters. One of these studies found that queens inseminated with frozen–thawed semen laid fertilized eggs for approximately six weeks, a significantly shorter duration compared to queens inseminated with fresh semen, which continued laying throughout the season [8]. One of our Fz colonies successfully entered wintering and, as of mid-October, remained productive, though it was weaker than the three F colonies also prepared for overwintering. Overall, 1 out of 5 Fz colonies entered wintering, consistent with a previous study where 9 out of 41 colonies were up for wintering [9]. A recent study reported that 13 out of 33 queens inseminated with frozen–thawed semen survived and produced female brood for over three months, with all 13 colonies successfully preparing for wintering [14]. It demonstrates the feasibility of obtaining strong colonies inseminated with frozen semen but also illustrates the room left for improvement in insemination with cryopreserved drone semen.

The limited egg-laying period for queens inseminated with frozen–thawed semen warrants further study. The proportion of colonies that successfully overwinter could serve as a valuable in vivo metric for assessing improvements in freezing protocols, as it likely reflects the number of sperm reaching the spermatheca and their long-term survival. It has been suggested that when less than 0.5 million sperms reach the spermatheca, queens face an increased risk of failing to lay fertilized eggs [12]. In our experimental design, the total inseminated sperm count in the Fz group was lower than in the F group. In order to preserve sperm motility and viability, semen was not centrifuged after thawing and reconcentrated [7]. Centrifugation, even at 250 RCF for 10 to 20 min, can damage sperm membranes [24]. Consequently, the fertility of queens from the Fz group may be affected both immediately and in the long term, likely shortening their egg-laying period. Cryoprotectant toxicity might also have impacted their female brood production.

Cryoprotectants, even though necessary to avoid the formation of ice crystals during slow freezing, show toxicity for the semen and the queen itself [23], and their post-thaw removal has been encouraged [21]. This might be performed by centrifugation or dialysis after thawing [9]. Alternatively, reducing the toxicity of cryoprotectants has been achieved by pre-freezing dialysis [10]. Unfortunately, these protocols add procedural complexity. Therefore, we opted against incorporating them to maintain protocol simplicity. The most commonly used cryoprotectant for drone semen freezing is DMSO, whose optimal concentration balancing both toxicity and protective effects is 10% [6,10,15,21,23], which was used in this study. A recent publication reported promising results with a higher final concentration of 12% cryoprotectants, a combination of polyvinylpyrrolidone (PVP) or Dextran, and DMSO. This formulation improved post-thaw motility and membrane integrity, as well as overwintering success, with 3 out of 13 queens still producing female brood 10 months after insemination [14]. This suggests that cryoprotectant removal might not be as necessary as previously proposed [23] and that higher concentrations might improve in vivo results. Additionally, a higher diluent-to-semen ratio has been used in other studies with promising results [11,14]. Combining this approach with increased dilution ratios to our simple protocol might also improve post-thaw quality and should be investigated.

In our protocol, the semen diluent composition shows a limited number of components to keep it as simple as possible, and no catalase was added. Catalase has been shown to improve post-thaw viability and maintain post-thaw motility when compared to other catalase-free diluents [11]. The positive impact of the adjunction of catalase on in vitro parameters has yet to be confirmed in fertility tests.

No antibiotics were added to the semen diluent, marking this as the first report of female offspring production from frozen–thawed semen without any antimicrobial addition. Research has confirmed that drone semen is not sterile [25], and antibiotics are often used to prevent bacterial growth, as a high bacterial load negatively impacts semen quality [26]. However, some antimicrobial properties may help manage Nosema apis, a sexually transmitted parasite [25]. Additionally, the use of antibiotics raises concerns about fostering antimicrobial resistance and potential impacts on queen health [19], so any inclusion should be carefully considered. The chosen semen collection method (aspiration via glass pipette) minimizes contamination compared to seminal vesicle dissection, which has shown a 100% contamination rate in one study. Although bacterial load did not impact in vitro sperm viability or motility in that study [25], it may have influenced queen survival in our experiment.

The queen mortality rate was high but comparable between the Fz and F groups, suggesting a common stress on the colonies. During the 2024 Belgian breeding season, beekeepers experienced high queen mortality following insemination, likely linked to the queens’ abnormally low weights. In our study, the average weight of the queens was 169.41 mg, whereas it is commonly accepted that a high-quality queen should weigh more than 200 mg after insemination. Queens with low weights, like those in our study, have been reported to experience lower acceptance rates and exhibit reduced sperm quantities in the spermatheca [27]. Semen quantity in the spermatheca is linked to the spermathecal volume, which is also linked to the weight of the queen [28]. The weight of a queen post-insemination is correlated with her weight at emergence [29], which is directly influenced by her rearing conditions, i.e., the age of the grafted larvae influences the weight at emergence [30]. Globally, queen-rearing success is influenced by available resources and environmental factors, such as humidity and temperature [31]. As all of these parameters were common to all F and Fz queens, it would have been interesting to multiply the number of queen breeders and test the effect of the queen-rearing impact on their survival. Additional environmental factors, such as ambient temperature, likely played a role, as three queens from the June batch appeared to die from overheating. At that time, we experienced a heat wave, and all of the bees nursing the queen were also found dead or dying. The other nuclei were moved to a cooler place before queen loss. This highlights the limitations of relying solely on in vivo assessments and highlights the need for reliable in vitro fertility metrics.

The highest post-thaw viability was observed in the first batch, which also exhibited the highest percentage of queens laying fertilized eggs. However, these queens did not sustain long-term female brood production and died within three weeks. Over 20 years ago, it was established that for a fixed inseminated volume of 8 µL of semen diluted 1:1, viability should reach at least 42% to produce only female brood, although female brood production has also been achieved with as low as 32% viable inseminated semen [32]. Unfortunately, in that study, semen concentration was not available, so the actual number of live sperm inseminated cannot be calculated. A fair estimation would be that around 40 million sperm were inseminated. This means that around 17 million live sperm cells would be necessary for producing exclusively female brood. Our results, partly agreeing with these estimations, suggest that it would be interesting to investigate the number of live sperm cells necessary to successfully produce female brood.

Overall, both methods used for viability assessment (CaV + PI and SYBR + PI) concurred. The main discrepancies were observed when less than 100 sperm cells could be evaluated. It is accepted that around 200 sperm cells should yield a precise evaluation of viability and that 100 cells should be a minimum [20]. Globally, the focus was more difficult to achieve when CaV was used in the fluorescent mix of dyes, rendering sample evaluation less effective for the same number of cells per microscopic field. To compensate, the number of acquired frames should have been increased. This could not be carried out as it prolongs light stimulation and thus increases photobleaching. As previously suggested, CaV associated with PI is efficient for evaluating viability but should most probably be used with flow cytometry instead of epifluorescence to take better advantage of this dye association. However, specific adjustments for drone semen and validation of the technique, with visual control under a microscope, are mandatory before the interpretation of results.

Overall, semen motility was low, characterized by minimal circular or active movement, with most sperm exhibiting linear position and vibratory motion. Various technical factors, such as chamber design and dilution parameters, can influence motility assessments, meaning results may vary under different conditions [33]. Recently, the CASABee software was developed to automatically analyze semen motility and concentration, thereby enhancing analytical accuracy [34]. Motility seems to be the best in vitro predictor of in vivo outcomes, such as sperm migration to the spermatheca and egg-hatching rates [6], although it may not correlate with overwintering success [14]. Automatic motility evaluation can be conducted in the field, requiring minimal technical expertise. Therefore, comparing manual observations with automatic measurements and in vivo metrics could have been useful in confirming motility as an accurate in vitro fertility predictor.

A primary limitation of this study is the low number of queens that initiated oviposition. Although the number of inseminated queens is comparable to those reported in other published protocols [7,8,9,14], statistical analyses were not conducted due to the limited number of queens that survived until oviposition. Nonetheless, the main objective of this study—testing the feasibility of obtaining female brood using a one-step antibiotic-free freezing protocol—has been successfully achieved.

## 5. Conclusions

This study highlights significant advancements in the use of frozen–thawed semen for honey bee insemination, particularly noting the potential for successful female brood production without the addition of antibiotics and the evaluation of viability by different combinations of dyes. While the observed outcomes demonstrate comparable queen mortality rates between frozen and fresh insemination techniques, the implications of low queen weight and limited egg-laying periods warrant further investigation. The findings emphasize the importance of refining cryopreservation protocols, including optimal cryoprotectant concentrations and minimizing contamination during semen collection, to enhance the viability and longevity of the sperm and also of the inseminated queens.

## Figures and Tables

**Table 1:** Semen analysis.

	Viability Mix 1 (%)	Viability Mix 2 (%)	Concentration (10^6^/µL)	Sperm Cells/Queen (10^6^)	Live Sperm Cells/Queen (10^6^)	Motility (%)
F 1	75 (184/246) *	76 (86/113)	6.4	32	24	15
Fz 1	77 (179/232)	56 (25/45)	4	20	15	7
F 2	86 (168/195)	87 (113/130)	9.4	47	40	5
Fz 2	39 (88/225)	33 (33/101)	4.2	21	8	1
F 3	95 (140/148)	79 (26/33)	15.4	77	73	2
Fz 3	29 (44/154)	10 (10/99)	6	30	9	1

* (Live sperm cells/total number of sperm cells evaluated).

**Table 2:** Insemination results.

Number of Queens That Survived Until:						
	Insemination	Release	Acceptation	Egg Laying	Female Brood	Overwintering Preparation
F 1	5	3	3	1	1	1
Fz 1	7	6	4	4	4	0
F 2	5	4	2	2	1	1
Fz 2	5	3	3	2	1	1
F 3	3	3	2	2	1	1
Fz 3	4	3	2	2	0	0
Total F	13	10	7	5	3	3
Total Fz	16	12	9	8	5	1

## Data Availability

Data are contained within the article.
